# Evaluation of Cerebrospinal Fluid Pressure by a Formula and Its Role in the Pathogenesis of Glaucoma

**DOI:** 10.1155/2019/1840481

**Published:** 2019-11-14

**Authors:** Laura Landi, Federica Casciaro, Serena Telani, Carlo E. Traverso, Alon Harris, Alice C. Verticchio Vercellin, Lauren Saint, Michele Iester

**Affiliations:** ^1^Clinica Oculistica, DiNOGMI, University of Genoa, Genoa, Italy; ^2^IRCCS Ospedale Policinico San Martino, Genoa, Italy; ^3^Department of Ophthalmology, Icahn School of Medicine, Mount Sinai Hospital, New York, NY, USA; ^4^University of Pavia, Eye Clinic, Pavia, Italy; ^5^IRCCS—Fondazione Bietti, Rome, Italy; ^6^Eugene and Marilyn Glick Eye Institute, Indiana University School of Medicine, Indianapolis, IN, USA

## Abstract

**Purpose:**

To investigate potential associations between intraocular pressure (IOP) and cerebrospinal fluid pressure (CSFP) in patients with primary open-angle glaucoma (POAG) and healthy subjects.

**Methods:**

Forty-three subjects were recruited. Weight and height were measured to calculate body mass index (BMI), along with blood pressure, heart rate, visual acuity, and IOP. Biometrics exam, corneal pachymetry, peripapillary retinal nerve fiber layer (RNFL) thickness, and macular thickness were assessed. The visual field exam was performed on all patients, and both pattern standard deviation (PSD) and mean deviation (MD) were considered. CSFP was estimated indirectly by using the mathematical formula CSFP = 0.44 × BMI + 0.16 × diastolic pressure − 0.18 × age − 1.91, based on the previous scientific studies. The TLCPD was calculated as follows: IOP−CSFP.

**Results:**

A significant (*p* < 0.05) difference was found between the two groups for several parameters. Specifically, the CSFP was lower in patients with POAG than in healthy subjects (8.14 ± 4.52 and 7.43 ± 2.06, *p* < 0.001, respectively). Anamnestic TLCPD was found to be significantly (*p* < 0.001) higher in patients with POAG compared to healthy subjects. A significant (*p* < 0.05) correlation was found between anamnestic TLCPD and MD (*r* = −0.31), inferior RNFL thickness (*r* = −0.29), superior RNFL thickness (*r* = −0.27), IOP (*r* = 0.22), and CSFP (*r* = −0.46).

**Conclusion:**

The CSFP was lower in glaucomatous patients compared to healthy subjects, whereas the TLCPD was higher in glaucomatous patients compared to healthy subjects, even though this difference was not statistically significant. A higher TLCPD may damage the RNFL, resulting in functional visual field impairment.

## 1. Introduction

The term glaucoma comprises a heterogeneous group of diseases with some characteristic features, including structural optic nerve head (ONH) damage and visual field loss. It has been proven that high intraocular pressure (IOP) is a risk factor for glaucoma, and that lowering IOP can decelerate disease progression [1]. However, increased IOP is not necessary for the development of glaucoma as the characteristic features of glaucoma may occur in susceptible subjects at any IOP [2]. This suggests that high IOP is not the sole risk factor for the development of glaucoma.

Other risk factors for glaucoma indicated in previous studies include age, decreased central corneal thickness, race, familial history of glaucoma, reduced ocular blood flow, low blood pressure, myopia, and lower ocular perfusion pressure [3–5]. Each risk factor is known to be associated with apoptotic processes that lead to glaucomatous optic neuropathy [6–9].

Many recent studies have reported that cerebrospinal fluid pressure (CSFP) and translamina cribrosa pressure difference (TLCPD) have a potential role in the pathogenesis of glaucomatous optic neuropathy [10–13]. The lamina cribrosa is positioned on the bottom of the scleral canal to close this hole, through which retinal ganglion cell axons exit the eye and form the ONH. Both the ONH and lamina cribrosa thus serve as barriers between intraocular and retrobulbar compartments. In the retrobulbar compartment, the optic nerve is surrounded by cerebrospinal fluid (CSF) in the subarachnoid space [11]. Therefore, on one side of the lamina cribrosa exists an IOP and on the other side of the laminar cribrosa exists a CSFP. The difference between these two pressures, IOP and CSFP, is the TLCPD. It has been postulated that a high TLCPD may damage and lead to abnormal functioning of the ONH due to changes in axonal transport and deformation of the lamina cribrosa [10–14].

Although many studies have shown that CSFP and the TLCPD seem to play a role in primary open-angle glaucoma (POAG), little is known about other parameters involved in the pathogenesis of glaucoma and those that may affect CSFP and TLCPD are age, blood pressure, body mass index (BMI), IOP, central corneal thickness (CCT), and structural and perimetric parameters [15].

The purpose of this study was therefore to evaluate the relationship between IOP and CSFP, as well as systemic and ocular parameters in healthy and glaucomatous subjects.

## 2. Materials and Methods

This research is a prospective study that has been approved by the Institutional Review Board of University Policlinic San Martino, Genoa, Italy, and was conducted in accordance with the principles of the Declaration of Helsinki. Patients joined the study after having signed a written informed consent. All subjects recruited for this study were selected at the Eye Clinic of University Policlinic San Martino, Genoa, Italy. All the clinical measurements were performed during the morning clinical session between 9.00 am and 12.00 am.

The study included forty-three subjects (26 POAG patients and 17 healthy subjects). All patients were questioned about their demographics, clinical history, ophthalmic history and ophthalmic medications, and systemic diseases and systemic medications. All participants underwent measurements of height (m), weight (kg), heart rate (HR) (bpm), and blood pressure (mmHg) in a standardized manner. Arterial hypertension was defined as a systolic blood pressure (SBP) > 140 mmHg, and/or a diastolic blood pressure (DBP) > 90 mmHg, and/or self-reported current treatment for arterial hypertension with antihypertensive medications. BMI was calculated using the formula body mass divided by the square of the body height and expressed in units of kg/m^2^.

The ophthalmic examination included measurements of visual acuity (VA), measurements of IOP using a calibrated Goldmann applanation tonometer, and slit-lamp examination of the anterior segment including gonioscopy and fundus. Biometric examination with IOLMaster (Carl Zeiss Meditec Inc., Dublin, California, USA) evaluated axial length (AL) (mm), anterior chamber depth (ACD) (mm), and corneal curvature (degrees). All patients underwent a spectral domain optical coherence tomography (SD-OCT) examination with RTVue SD-OCT (Optovue, Inc., Fremont, California, USA), in order to evaluate the thickness of the peripapillary retinal nerve fiber layer (RNFL), the foveal thickness (microns), and CCT (microns). The Humphrey 24-2 Swedish interactive threshold algorithm standard perimeter (Carl Zeiss Meditec Inc., Dublin, California, USA) was used to evaluate the visual field. To estimate the CSFP, a formula cited in Xie et al.'s study was used: CSFP = BMI × 0.44 + 0.16 × diastolic BP − 0.18 × AGE − 1.91 [15–17]. When this formula was tested on an independent study group, it revealed that the measured CSFP did not differ significantly (*p*=0.29) from the calculated CSFP (12.6 mmHg vs. 13.3 mmHg, respectively); [15–17] however, the formula hypothesized that the orbital subarachnoid space width, as measured by orbital MRI, can be used to estimate the CSFP.

Furthermore, the TLCPD was calculated as IOP−CSFP. Both current and anamnestic TLCPD were calculated by using the data on the patients' files. For the anamnestic data, we used the information collected at the first visit, and when missed, we used younger data found in the file, but not the data of the last visit.

The recruited participants were classified into two subsets: POAG patients (*n* = 26) and healthy subjects (*n* = 17).

POAG eyes were diagnosed based upon having a reproducible and characteristic visual field defect of 3 nonedge points which were all depressed on the pattern deviation plot at *p* < 5%, along with an asymmetrical cupping greater than 0.2, the presence of a notch on the rim, and/or an increased cupping greater than 0.6 at fundoscopy. Reliable tests had fewer than 20% fixation losses and less than 15% false-negative and false-positive responses.

Healthy subjects had normal visual field and optic disc in accordance with IOP <21 mmHg.

Exclusion criteria included history of active or past ophthalmological diseases other than glaucoma such as uveitis, maculopathy, corneal abnormalities (keratoconus or corneal dystrophies), any history or slit-lamp evidence of angle-closure glaucoma, history of ocular surgery or laser treatments, history of ocular trauma, use of systemic steroids, any other systemic medication known to affect the retina, any neurological condition known to affect the visual field, and previous history of brain surgery that would affect the pressure and structural changes of the optic nerve.

## 3. Statistical Analysis

The data were evaluated by descriptive analysis. When the distribution of the data was normal, a two-tailed paired *t*-test was used. When the distribution of the data was nonnormal, a Mann–Whitney test was used. Pearson correlation coefficient was used to analyze the correlations among all the parameters. A *p* value <0.05 was considered statistically significant. A multivariate analysis was performed to verify the effect of age, gender, and BMI on the correlation studied.

## 4. Results

The descriptive parameters measured in the POAG and healthy group are summarized in Table 1. Of the 26 patients enrolled with a diagnosis of POAG, 12 were female and 14 were male, aged between 54 and 84 years (mean average age: 72.3 ± 7.7 years). The results obtained were compared with 17 healthy subjects, 8 female and 9 male, aged between 42 and 89 (mean average age: 68.1 ± 15.6 years).

In the POAG group, 18 patients were suffering from high blood pressure treated pharmacologically and one of these patients was also suffering from noninsulin dependent diabetes mellitus (NIDDM) treated with glibenclamide.

In the healthy group, one subject had a positive family history of glaucoma and 8 subjects were suffering from high blood pressure treated pharmacologically. Of these, 4 were suffering from cataracts (2 right eyes and 2 left eyes) and one was suffering from NIDDM. Of the healthy patients without hypertension or positive family history of glaucoma, 6 were affected by cataracts solely in the left eye, and one patient had previously had an acute attack of glaucoma in the right eye, so we considered only the left one. Lastly, another nonhypertensive healthy patient without positive family history of glaucoma was suffering from NIDDM treated with glibenclamide.

As shown in Table 1, the parameters that differ significantly between the glaucoma and healthy groups were age, CSFP, anamnestic IOP, anamnestic TLCPD, MD, and PSD (*p* < 0.001); VA, refractive error, and superior RNFL thickness (*p*=0.001); weight (*p* value between 0.001 and 0.01); and height and inferior RNFL thickness (*p* value between 0.01 and 0.05).

Tables 2 and 3 summarize Pearson's *r* correlation coefficients performed to assess possible correlations between all considered parameters. When a multivariate analysis was performed, age, gender, and BMI did not have significant effect on the considered parameters such as MD, PSD, RNFL, foveal thickness, and TLCPD.

## 5. Discussion

Discussions about CSFP's importance to the ONH and, in particular, its potential role in the pathogenesis of glaucomatous optic neuropathy have been ongoing for 40 years, beginning with Volkov in 1976 [18], followed by Morgan et al. in 1995 [19] and many others. Recently, Ren et al. [20] found that in open-angle glaucoma with normal IOP, CSFP is abnormally low, leading to an abnormally high translamina cribrosa pressure difference. They suggested that a low CSFP in normal-IOP glaucoma may be similar to a high IOP in high-IOP glaucoma. Depending on the posture, the average IOP is higher than the average CSFP, impinging on the TLCPD. A normal IOP with a low CSFP results in the same TLCPD as patients who have an elevated IOP but normal CSFP [21]. The pressure gradient through the lamina cribrosa can be defined as the distance between the intraocular compartment and the retrobulbar one, that is, the thickness of the lamina cribrosa [10]. It has been shown that the cribriform plate is thinner in patients who have glaucomatous damage of the optic nerve. However, curiously, experimental studies have also found that the cribriform plate is thicker in the early stages of glaucoma [11, 12]. The changes of the TLCPD may explain the altered function and optic nerve damage in relation to changes in axonal transport and deformation of the lamina cribrosa in glaucoma patients.

The aim of our study was to investigate the relationship between CSFP and IOP, as well as other systemic and ocular parameters in healthy and glaucomatous subjects. Patients with POAG were found to have a statistically lower CSFP compared to healthy patients (*p* < 0.001, Table 1). Both the current and anamnestic TLCPD were higher in glaucoma patients compared to healthy controls, but while the difference of TLCPD was not significant (*p*=0.235), anamnestic TLCPD was statistically significant (*p* < 0.001, Table 1). This could be due to the IOP values used: indeed when no significant difference was found, the IOP measured was under topical treatment. There are numerous studies in the literature that deal with the relationship between CSFP, TLCPD, and IOP and their direct consequences on the physiology of the eye. In 2015, Jonas et al. outlined that the cerebrospinal fluid space extends from the intracranial compartment, through the tiny optic canal, and ends posterior to the eyeball [9]. Therefore, the pressure of the orbital portion of the optic nerve is not equal to the real orbital pressure (about 2 mmHg), but is at least as high as the CSFP. This anatomical relationship could have profound importance for the physiology and pathophysiology of the optic disc, which acts as a barrier between the intraocular and the retrobulbar compartments. The TLCPD, which is determined by the difference between the IOP and CSFP, is known to play a direct role in the damage of the optic nerve head [9]. In 2008, the retrospective study of Berdahl et al. showed the CSFP of glaucomatous and nonglaucomatous patients who underwent lumbar puncture [13], and the authors found that CSFP was significantly lower in the group of patients with glaucoma compared to the nonglaucomatous control group. Another larger retrospective study performed by Berdahl et al. in 2008 upheld the results of their previous study, but also found that patients with ocular hypertension had a significantly elevated intracranial pressure compared to the control group [22].

Although the studies from Berdahl et al. were limited by small samples of patients, their findings suggested that glaucomatous optic neuropathy was associated with low CSFP, while ocular hypertension was associated with high CSFP [13, 22]. Other studies, like the Beijing Eye Study [14] and Central India Eye Study [23], instead of directly measuring CSFP, estimated it by applying the formula developed by Xie et al. in 2013 (CSFP = 0.44 × BMI + 0.16 × diastolic pressure − 0.18 × age − 1.91), as we did in our study [15]. Similarly, in 2016, Siaudvytyte et al., utilizing two-depth transcranial Doppler technology to noninvasively measure CSFP and ocular perfusion pressure in patients with normal tension glaucoma (NTG), found that patients with lower intracranial pressure (ICP) had both more pronounced structural damage and worsened ocular hemodynamic levels [24]. The same authors examined the differences in the TLCPD and neuroretinal rim area (NRA) between patients with NTG, with high-tension glaucoma, and healthy subjects and found that there was a correlation between NRA and TLCPD solely in the NTG group (*r* = −0.83, *p*=0.01), which confirmed the idea that decreased ICP produces increased TLCPD and leads to glaucomatous damage [25]. These studies, despite their wide geographical, cultural, social, and economic differences of the studied subjects, uphold previous results and the results of our study, demonstrating that patients with glaucoma have a lower estimated CSFP.

As shown in Table 1, compared to healthy subjects, POAG patients also differ significantly in age, IOP, RNFL thickness, PSD, height, and weight (all *p* values <0.05, Table 1), but these differences were not active on the correlation as multivariant analysis shows. Concerning the association between measurements, we found a significant association (all *p* values <0.05, Tables 2 and 3) between anamnestic TLCPD and weight (*r* = −0.299), superior (*r* = −0.273) and inferior (*r* = −0.294) RNFL thickness, and MD (*r* = −0.319) (Tables 2 and 3) and other parameters which were included in the formula such as age (*r* = 0.269), BMI (*r* = −0.356), IOP (*r* = 0.223), and CSFP (*r* = −0.456). Importantly, the negative correlation between anamnestic TLCPD and RNFL thickness agrees with the results previously reported by Siaudvytyte et al. [25] who found a negative correlation between NRA and TLCPD in NTG patients. These data could suggest a possible relationship between TLCPD and the loss of ganglion cells evaluated as RNFL thickness. It is also important the correlation between anamnestic TLCPD and MD, which outlined the possible relationship with the ONH function detected by perimetry.

These results are particularly significant when considering the data from the literature concerning the well-known risk factors for glaucoma onset and progression. The literature consistently shows that increasing IOP and age are the major risk factors for the development and progression of glaucoma [1]. Similarly, the RNFL is consistently thinner and PSD higher among glaucoma patients and these two measurements represent strong diagnostic structural and functional tools for the disease [26]. The literature demonstrates conflicting results regarding the relationship between BMI and glaucoma [27, 28]. In our study, however, a significant correlation was found between BMI and anamnestic TLCPD (Table 2). Taken together, our findings suggested that glaucomatous optic nerve damage can be the result of a complex relationship between several risk factors, which are all associated with TLCPD in our study.

Our results have two main limitations: Firstly, the sample size of our study was small (53 eyes of 26 patients with POAG and 33 eyes of 17 healthy subjects) and consists solely of Caucasian patients. The results may not be transferable across all populations, and further studies should be conducted with larger sample sizes. Moreover, we also used a calculation to indirectly calculate the CSFP, which may not equal the true orbital CSFP [23]. However, unlike previous studies regarding CSFP and glaucoma as in Berdahl et al.'s retrospective studies, our study is a prospective one, which limits the potential for confounding variables and biases [13, 22].

In conclusion, this study showed that the estimated CSFP was significantly lower in POAG patients compared to the control group, whereas the TLCDP in POAG patients was higher, even if the difference was not statistically significant, than in the control group, but clinically, the data of anamnestic TLCDP were more useful and showed both significant difference between the two groups and good correlation with other clinical parameters. These results agree with previous studies. The CSFP may lead to stress on the peripapillary RNFL, as demonstrated by the RNFL values. As a result, patients will have corresponding functional damage, detectable by the values of MD and PSD in perimetry. Therefore, our findings suggested that orbital CSFP was a counter-pressure to IOP and was an important determinant of TLCPD, the pathophysiology and physiology of the optic nerve head, and pressure-related diseases, such as glaucomatous optic neuropathy.

## Figures and Tables

**Table 1:** Descriptive statistics for glaucomatous and nonglaucomatous patients.

	Glaucoma (*n* = 53)	Normal (*n* = 33)			Comparison test
	Mean	Standard deviation	Mean	Standard deviation	*p* value
Age (years)	72.32	7.67	68.13	15.59	<**0.001**
Systolic blood pressure (mmHg)	124.91	15.24	130	16.22	0.986
Diastolic blood pressure (mmHg)	71.32	9.10	71.88	9.81	0.425
Height (m)	1.65	0.09	1.68	0.11	**0.024**
Weight (Kg)	67.45	10.98	70.69	21.57	**0.004**
BMI (kg/m^2^)	24.87	3.17	24.58	5.32	0.085
Heart rate (bpm)	66.26	11.04	66.88	11.02	0.745
Visual acuity (10/10)	9.4	1.43	8.12	2.29	**0.001**
Refractive error (diopters)	1.22	1.81	−3	5.196	**0.001**
IOP (mmHg)	15.85	2.73	15.18	1.62	0.208
Anamnestic IOP (mmHg)	21.04	5.71	15	1.97	<**0.001**
CSFP (mmHg)	7.43	2.06	8.14	4.52	<**0.001**
Axial length (mm)	23.68	1.58	24.09	1.99	0.542
Anterior chamber depth (mm)	2.76	0.78	2.49	0.58	0.585
CCT (um)	534.21	36.09	552.69	30.96	0.210
Superior RNFL thickness (um)	83.06	13.29	94.75	6.05	**0.001**
Inferior RNFL thickness (um)	79.83	13.97	91.63	7.68	**0.013**
Foveal thickness (um)	253.11	29.06	272.69	30.15	0.862
MD (dB)	−3.59	4.72	−0.47	0.72	<**0.001**
PSD (dB)	4.32	4.33	1.57	0.43	<**0.001**
TLCPD (mmHg)	8.42	3.27	7.51	3.47	0.235
Anamnestic TLCPD (mmHg)	13.61	6.18	7.33	3.97	<**0.001**

*n* = number of eye; BMI = body mass index; IOP = intraocular pressure; CSFP = cerebrospinal fluid pressure; CCT = central corneal thickness; RNFL = retinal nerve fiber layer; MD = mean deviation; PSD = pattern standard deviation; TLCPD = translamina cribrosa pressure difference.

**Table 2:** Pearson's correlation coefficients (*r*) values.

	CSFP	TLCPD	Anamnestic TLCPD	AL	ACD	Foveal thickness	CCT
Age	−**0.653**^*∗∗*^	**0.471** ^*∗∗*^	**0.269** ^*∗*^	−**0.289**^*∗∗*^	0.063	−0.138	−0.015
Systolic BP	**0.317** ^*∗∗*^	−0.093	−0.161	−0.127	−**0.430**^*∗∗*^	**0.241** ^*∗*^	0.128
Diastolic BP	**0.501** ^*∗∗*^	−**0.229**^*∗*^	−0.134	−0.068	−**0.308**^*∗∗*^	**0.345** ^*∗∗*^	−0.106
Height	0.177	−0.072	−0.065	**0.456** ^*∗∗∗*^	**0.259** ^*∗*^	0.041	0.046
Weight	**0.487** ^*∗∗*^	−**0.399**^*∗∗*^	−**0.299**^*∗∗*^	**0.240** ^*∗*^	0.094	−0.163	**0.234** ^*∗*^
BMI	**0.530** ^*∗∗*^	−**0.489**^*∗∗*^	−**0.356**^*∗∗*^	−0.044	−0.116	−**0.258**^*∗*^	**0.291** ^*∗∗*^

^*∗*^Significant *p* value <0.05, and ^*∗∗*^significant *p* value ≤0.01. BP = blood pressure; BMI = body mass index; CSFP = cerebrospinal fluid pressure; TLCPD = translamina cribrosa pressure difference; AL = axial length; ACD = anterior chamber depth; CCT = central corneal thickness.

**Table 3:** Pearson's correlation coefficients (*r*) values.

	CSFP	TLCPD	Anamnestic TLCPD	SUP RNFLT	INF RNFLT	MD	PSD
IOP	0.131	**0.600** ^*∗∗*^	**0.223** ^*∗*^	0.065	−0.103	0.130	−0.147
CSFP	1	−**0.714**^*∗∗*^	−**0.456**^*∗∗*^	0.097	0.154	0.075	−0.109
TLCPD	−**0.714**^*∗∗*^	1	0.526^*∗∗*^	−0.031	−0.196	0.051	−0.039
Anamnestic TLCPD	−**0.456**^*∗∗*^	0.526^*∗∗*^	1	−**0.273**^*∗*^	−**0.294**^*∗∗*^	−**0.319**^*∗*^	0.218
SUP RNFLT	0.097	−0.031	−**0.273**^*∗*^	1	**0.715** ^*∗∗*^	**0.446** ^*∗∗*^	−**0.466**^*∗∗*^
INF RNFLT	0.154	−0.196	−**0.294**^*∗∗*^	**0.715** ^*∗∗*^	1	**0.390** ^*∗∗*^	−**0.462**^*∗∗*^

^*∗*^Significant *p* value < 0.05, and ^*∗∗*^significant *p* value ≤0.01. IOP = intraocular pressure; CSFP = cerebrospinal fluid pressure; TLCPD = translamina cribrosa pressure difference; SUP RNFLT = superior retinal nerve fiber layer thickness; INF RNFLT = inferior retinal nerve fiber layer thickness; MD = mean deviation; PSD = pattern standard deviation.

## Data Availability

The data used to support the findings of this study are available from the corresponding author upon request (iester@unige.it).
